# Motion-triggered video cameras reveal spatial and temporal patterns of red fox foraging on carrion provided by mountain lions

**DOI:** 10.7717/peerj.5324

**Published:** 2018-07-31

**Authors:** Connor O’Malley, L. Mark Elbroch, Patrick E. Lendrum, Howard Quigley

**Affiliations:** 1 Panthera, New York, NY, USA; 2 Department of Fish, Wildlife and Conservation Biology, Colorado State University, Fort Collins, CO, USA

**Keywords:** *Vulpes vulpes*, *Puma concolor*, Carrion, Scavenging, Apex predator, Trophic cascade, Motion-triggered video cameras, Mountain lion, Red fox

## Abstract

Carrion is a rich, ephemeral resource vital to biodiversity and ecosystem health. In temperate ecosystems in which cold temperatures and snowfall influence the accessibility and availability of small prey and seasonal mast crops, carrion may also be a limiting resource for mesocarnivores like red foxes (*Vulpes vulpes*), which are too small to predate ungulates. Using motion-triggered video cameras and generalized linear mixed models, we studied the spatial and temporal patterns of red fox scavenging at 232 mountain lion kills in the southern Greater Yellowstone Ecosystem (GYE) from 2012–2015. We found that red foxes scavenged mountain lion kills across all habitats throughout the year, however, red fox behaviors varied with season. In winter, we documented red foxes at a greater proportion of mountain lion kills (70.3% in winter vs. 48.9% in summer), and in greater numbers (1.83 foxes per kill in winter vs. 1.16 in summer). In winter, red foxes fed longer (= 102.7 ± 138.3 minutes feeding in winter vs. = 39.7 ± 74.0 in summer), and they more often scavenged while the mountain lion was nearby. We speculated that red foxes may have increased risk taking in winter due to hunger driven by resource scarcity. Our research highlighted an important ecological relationship between red foxes and mountain lions in the GYE. Mountain lions tolerate high levels of scavenging, so the frequency and intensity of red fox scavenging at their kills may not impact mountain lions, but instead facilitate the dispersion and benefits of resources created by this apex predator. Large carnivores, and mid-trophic felids like mountain lions in particular, are essential producers of carrion vital to biodiversity and ecosystem health. In turn, scavengers play critical roles in distributing these resources and increasing the heterogeneity of resources that support biodiversity and ecosystem structure, as well as ecological resilience.

## Introduction

Carrion is a rich, ephemeral resource exploited by diverse vertebrate and invertebrate scavengers and decomposers (Wilson & Wolkovich, 2011; Moleón & Sánchez-Zapata, 2015). Carrion is also a dependable resource for mesocarnivores, such as red foxes (*Vulpes vulpes*), where large carnivores live at sufficient densities to support the wide-spread distribution of carrion resources (Wilmers et al., 2003b; Selva et al., 2005; Sivy et al., 2017). Red foxes exhibit the largest geographic range of any member of the Order Carnivora, inhabiting 70 million km^2^ across 89 countries (Feldhamer, 1987), across which they display tremendous adaptability in spatial ecology and density, social plasticity, and foraging ecology (Doncaster & Macdonald, 1991; Cavallini, 1996; Walton et al., 2017). For example, red foxes are quick to exploit novel foods, even when there are substantial risks associated with foraging for them (e.g., Wilmers et al., 2003a; Contesse et al., 2004; Helldin & Danielsson, 2007).

Red fox populations exhibit wide dietary breadth, eating small vertebrates and plant matter, as well as scavenging carcasses of prey killed by larger carnivores that could kill them (Helldin & Danielsson, 2007; Elbroch et al., 2017; Sivy et al., 2017). Following the recolonization of Eurasian lynx (*Lynx lynx*) in Sweden, for example, red foxes switched their diet from one dominated by fish, reptiles, invertebrates and vegetables to one dominated by lynx-killed roe deer (Helldin & Danielsson, 2007). Red foxes are also known to scavenge at the kills of mountain lions (*Puma concolor*; Elbroch et al., 2017) and gray wolves (*Canis lupis*; Wilmers et al., 2003a; Selva et al., 2005), and likely scavenge from the kills of other carnivores with which they are sympatric.

In temperate ecosystems in which snowfall influences the accessibility and availability of small prey (Sargeant, Allen & Eberhardt, 1984; Halpin & Bissonette, 1988) and mast crops (Cross, 2015), carrion may be a limiting resource for red foxes, which are too small to predate on ungulates (Selva et al., 2005; Pereira, Owen-Smith & Moleón, 2014). Scavenging likely increases fitness for red foxes as has been shown for other animals. Arctic foxes (*Vulpes lagopus*), for example, increase their utilization of carrion provided by polar bears (*Ursus maritimus*) in times when lemmings (*Lemmini spp.*) are scarce (Roth, 2003).

**Figure 1:**
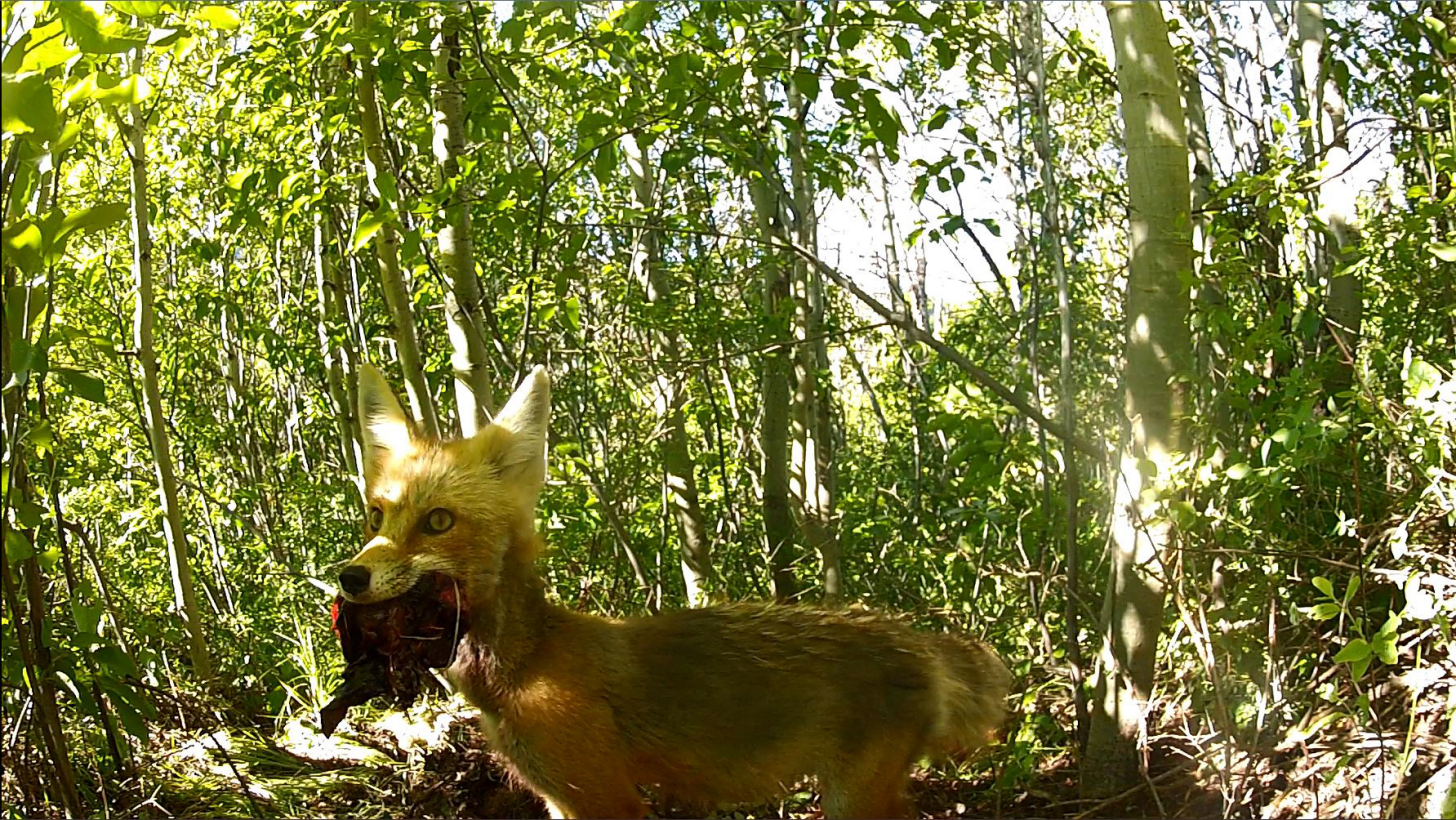
A red fox carries away a large portion of mule deer carcass, a summer prey killed by an adult female mountain lion in the southern Greater Yellowstone Ecosystem. Red fox caching aids in dispersing carrion resources, increasing the contributions of this apex predator to its larger ecological community. Photograph by L Mark Elbroch.

Simultaneously, scavenging by red foxes likely benefits their broader ecological communities. Scavengers disperse carrion nutrients away from carcasses through feeding and caching (Fig. 1), increasing the heterogeneity of resources that support biodiversity and ecosystem structure (Bump, Peterson & Vucetich, 2009; Cortés-Avizanda et al., 2009; Barton et al., 2013; Allen et al., 2015). Scavenging also increases food web linkages that bolster ecological resilience (DeVault, Rhodes Jr & Shivik, 2003; Wilson & Wolkovich, 2011; Moleón et al., 2014).

Using motion-triggered video cameras, we studied the temporal and spatial patterns of red fox scavenging at mountain lion kills in the southern Greater Yellowstone Ecosystem (GYE) to better understand how foxes utilize carrion and benefit from top predators. More broadly, we were interested in studying the potential tradeoffs faced by foxes with regards to seasonal food availability and intraguild predation. With regards to temporal patterns, we hypothesized that foxes would utilize carrion more often in winter for two reasons: (1) the availability of other seasonal food sources (e.g., small mammals, migratory waterfowl, mast crops) was reduced (Jedrzejewski & Jedrzejewska, 1992; Sidorovich, Sidorovich & Izotova, 2006; Pereira, Owen-Smith & Moleón, 2014), (2) mountain lion kill sites were aggregated in areas where wintering elk herds congregated near supplementary feed (Elbroch et al., 2013), and subordinate competitors more successfully exploit carrion resources when carrion is aggregated (Wilmers et al., 2003a). Specifically, we predicted that in winter, red foxes would more often be detected at mountain lion kills, would feed longer at carcasses, and that there would be a greater number of foxes at each carcass in winter when compared with summer. Because mountain lions limited gray fox scavenging in a Mediterranean system (Allen et al., 2015), we also predicted that red foxes would wait to exploit carrion at mountain lion kills until after the carcass was abandoned by the mountain lion that made the kill. In the instances when they fed while the mountain lion was still in residence, we predicted that foxes would exhibit higher vigilance behaviors to mitigate predation risk while the mountain lion was near the carcass, than after the mountain lion had departed the site. In terms of spatial distribution, we hypothesized that red foxes would preferentially scavenge at mountain lion kill sites throughout the year near forest edges or in structured habitats, as these habitats would again mitigate predation risk and offer foxes protection from mountain lions as well as other carnivores during encounter competition for carrion resources. We predicted that foxes would not show a preference for kills in relation to elevation, aspect or terrain ruggedness.

**Figure 2:**
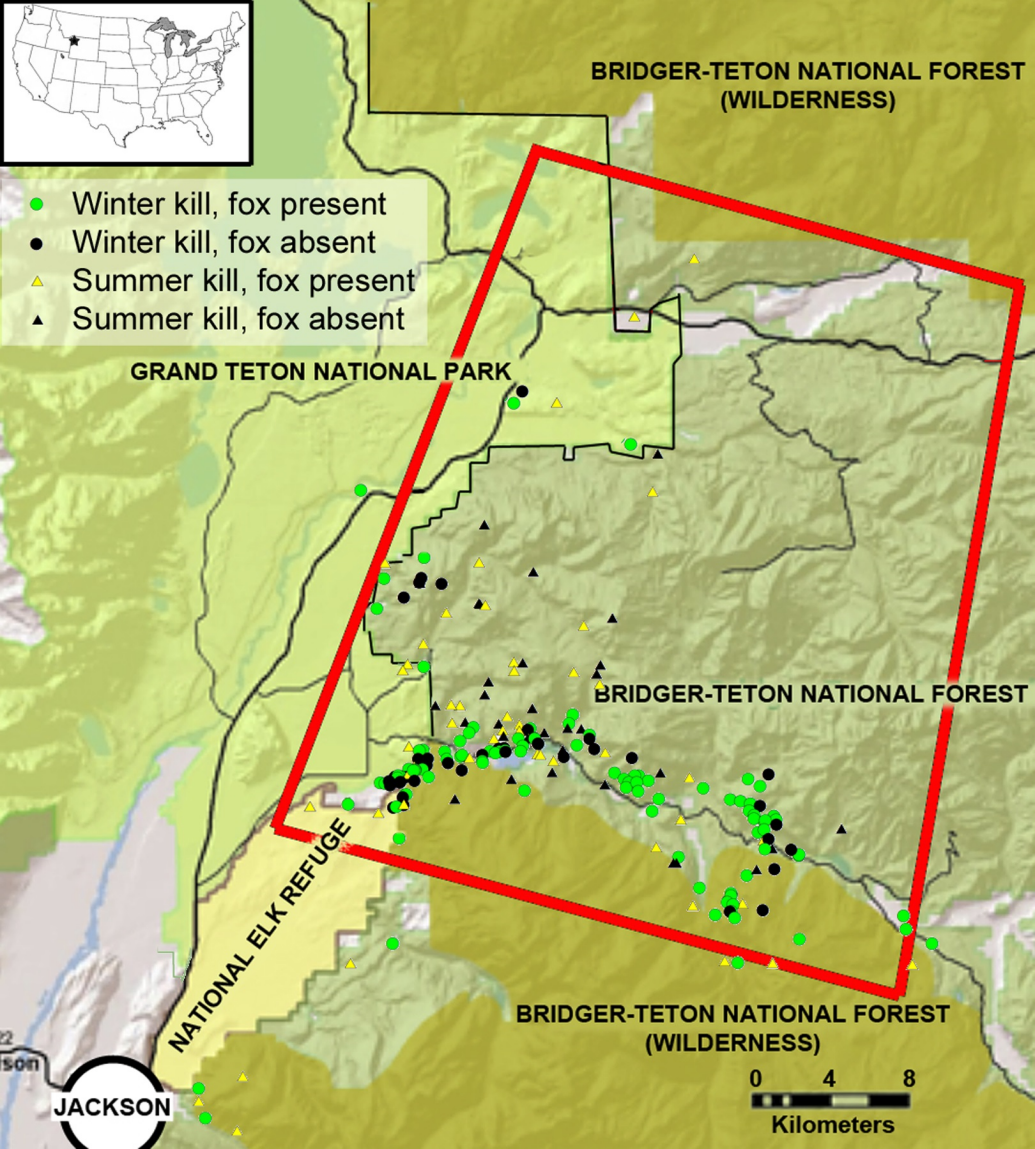
The study area in the southern Greater Yellowstone Ecosystem, Teton County, Wyoming. The inset map depicts the location in the lower United States, and the red rectangle highlights our primary research area in which we followed mountain lions. Icons represent winter and summer mountain lion kills sampled for this research, and at which kills red foxes were present. Study area map created and provided by L Mark Elbroch.

## Methods

### Study area

Our study area covered approximately 2,300 km^2^ in the southern GYE north of Jackson, Wyoming (Fig. 2). Our research was conducted on Bridger-Teton National Forest (United States Forest Service, USFS Authorization ID JAC760804), Grand Teton National Park (NPS Permit GRTE-2012-SCI-0067), and National Elk Refuge (USFW permit NER12). Elevations ranged from 1,800 m to >3,600 m contributing to long winters with high snow pack. The terrain was characterized by glacially scoured sagebrush valleys, densely forested north-facing slopes, and uplifted south facing cliffs with abundant evidence of landslides. Ungulate prey such as pronghorn (*Antilocapra americana*) and mule deer (*Odocoileus hemionus*) migrated out of the study area during winter, while elk (*Cervus elaphus*) remained and aggregated in lower elevations where they received supplemental feed (Jones et al., 2014). Red fox competitors for carrion resources included mountain lions, black bears (*Ursus americanus*), grizzly bears (*Ursus arctos horribilis*), and gray wolves, as well as coyotes (*Canis latrans*), bald eagles (*Haliaeetus leucocephalus*), golden eagles (*Aquila chrysaetos*), ravens (*Corvus corax*) and black-billed magpies (*Pica pica*). Potential prey species for foxes in our study area included voles (*Microtus spp.*), northern pocket gophers (*Thomomys talpoides*), deer mice (*Peromyscus maniculatus*), snowshoe hares (*Lepus americanus*), chipmunks (*Tamias spp.*), Uinta ground squirrels (*Spermophilus armatus*), ruffed grouse (*Bonasa umbellus*), and dusky grouse (*Dendragapus obscurus*). The availability of small mammals varied seasonally, as some species hibernated (chipmunks and ground squirrels) and others were influenced by accessibility determined by snow and ice pack. Red foxes also consume invertebrates (Macdonald, 1977) and seeds from whitebark pine (*Pinus albicaulis*; Cross, 2015), which were also present in our study area. Further details about the flora and fauna of the study area are found in Elbroch et al. (2013).

### Mountain lion capture and collar programming

Our mountain lion capture protocols followed guidelines outlined by the American Society of Mammalogists (Sikes et al., 2011), as previously described in Elbroch et al. (2013) and were approved by two independent Institutional Animal Care and Use Committees (IACUC): the Jackson IACUC (Protocol 027-10EGDBS-060210) and National Park Service IACUC (IMR_GRTE_Elbroch_Cougar_2013-2015), with permission to handle cougars granted by the Wyoming Game and Fish Department (Permit 297). We captured mountain lions with baited cage traps and scent-trailing hounds from 2012–2015. Captures took place during winter months when snow facilitated the detection of mountain lions by researchers and cooler temperatures created safer animal handling conditions. Mountain lions were immobilized with ketamine (4.0 mg/kg) and medetomidine (0.07 mg/kg), and their vital signs were monitored at 5-min intervals. We fit them with a GPS collar (Lotek Globalstar S or Iridium M, Newmarket, Ontario; Vectronics Globalstar GPS Plus, Berlin, Germany), and the effects of the capture drugs were reversed with Atipamezole (0.375 mg/kg). We remained with the mountain lions until they were mobile and then we observed their departure from the capture site.

### Data collection at mountain lion kills

CyberTracker-certified researchers (Evans et al., 2009; Elbroch et al., 2011) conducted site investigations of aggregated GPS locations, or “clusters” (Anderson Jr & Lindzey, 2003), in the field, as previously described by Elbroch et al. (2014). We defined clusters as two or more GPS locations within 150 m of each other, at least 4 hrs in duration and at most, 2 weeks apart; we included clusters made at any time of day or night. When we discovered prey during site investigations, we judged whether there was sufficient meat to draw in scavengers (Elbroch et al., 2017). If so, we placed paired motion-triggered cameras (Bushnell Outdoor Products, Overland Park, KS, USA) with 32 GB cards to record activity at the kill. Cameras were programmed to record 60 s videos with 30 s delay intervals, and they gathered data at the carcass for varying lengths of time, dependent upon the availability of carrion, whether the carcass was moved, and the battery life of the camera (Elbroch et al., 2017). Between 2012 and 2015 we placed motion triggered cameras at 232 mountain lion kill sites of prey >25 kg, 138 during the winter months and 94 during the summer.

### Red fox behavior analyses

We cataloged each video collected at mountain lion kills in an Access database, recording species present, species interactions, vocalizations, and other behaviors (e.g., feeding, resting, moving). For each kill site, we determined whether foxes were detected, confirmed that they had indeed scavenged the carcass, and also estimated the number of foxes at the carcass. As we were unable to reliably differentiate between individuals, especially from coarse black and white footage obtained at night, we defined the minimum number of foxes at each kill site as the maximum number of individuals counted in any video from the kill in the same frame (i.e., at the same time). We estimated “total” fox feeding time by summing the 60 s videos in which one or more foxes were visibly feeding on the carcass. We classified behaviors such as looking, listening, and moving (gaining a vantage point to investigate a sound or sight) as vigilance behavior.

We compiled all data and conducted all our analyses across two seasons, summer (May–October) and winter (November–April), based on typical weather patterns of the region (SNOTEL weather station 506), and the influence that weather has on food availability for foxes and mountain lion foraging behaviors. First, we tested whether there was a difference in the proportion of mountain lion kills at which foxes were detected scavenging in summer versus winter with a two-proportions *z*-test, and then we compared the mean number of foxes detected at kills in the two seasons with a *t*-test.

We determined the mountain lion’s departure time and date from video data, and confirmed it with GPS data collected from collars. We separated the data into summer and winter, as detailed in ‘Spatial analyses to determine the distribution of red fox scavenging’. Then we compared red fox presence before and after the mountain lion’s departure (binary data) with a McNemar test, a variation of a contingency test that can compare paired categorical rather than continuous data, to determine whether foxes were waiting for mountain lions to leave before scavenging.

We classified a mountain lion as “in residence” from the time that the kill was made, as determined with GPS data, to the final feeding bout at the carcass, as determined by video footage and GPS data. In cases when red foxes fed while mountain lions were in residence, we also analyzed fox vigilance behavior. To estimate vigilance, we divided the number of 60 s videos in which red foxes were feeding by the total number of videos in which red foxes were recorded exhibiting any behavior (e.g., feeding + moving, watching, listening). Then we used a *t*-test to determine whether foxes exhibited different proportions of their time feeding at the carcass while the mountain lion was still feeding from the carcass versus after it had departed.

### Spatial analyses to determine the distribution of red fox scavenging

Although it was well documented that prey aggregate in winter versus summer in our study system (Jones et al., 2014), we conducted simple nearest neighbor analyses in ArcGIS to determine whether the mountain lion kills we sampled with cameras were also more clustered in winter than summer. We did this for kills in the southern half of our study area (southern half of red rectangle in Fig. 2), where we had the most data. We compared these distances with a *t*-test to determine whether they were statistically different.

Then we identified five landscape variables as potentially important predictors of where foxes were likely to occur at mountain lion kill sites: elevation (m), aspect (transformed into categories of North, East, South, West), terrain ruggedness (vector ruggedness measure; VRM), habitat type, and distance to forest edge. We estimated elevation using a digital-elevation model (DEM) at a resolution of 30 m (http://datagateway.nrcs.usda.gov/). We then used ArcGIS 10.0 Spatial Analyst Tools to derive values of slope and aspect from the DEM. In addition, we also derived a vector ruggedness measure (VRM) from the DEM (following Sappington, Longshore & Thompson, 2007). A Gap Analysis Program (GAP) land cover (gapanalysis.usgs.gov/gaplandcover) was used, at a resolution of 30 m, which included 87 cover classes, which we reclassified into four cover classes based on similarity of land cover types: (1) open meadows, (2) shrub-steppe, (3) forested, (4) and riparian zones. We converted all forested lands from the GAP into a polygon layer in ArcGIS to and then created edge layers as the perimeter of each forested section. All distance variables were standardized by subtracting their means and dividing by their standard deviations, to aid in model convergence and for direct comparison of variables (Gelman & Hill, 2006).

We conducted our analyses for two seasons, as described above. We examined the relationship between mountain lion predation sites (following methods in Elbroch et al., 2013) and where foxes scavenged at predation sites by comparing known kill sites where foxes were documented to kill sites where they were absent. To do so, we used generalized linear mixed models that allowed for random effects to control for variation among samples. We included the number of days a camera recorded videos while a carcass was present as a random intercept to account for sampling bias that may have resulted from different sampling intervals (Elbroch et al., 2017).

Prior to modeling, we used a correlation matrix to evaluate collinearity (—*r*— >  0.6) among predictor variables. No predictor variables were correlated (—*r*— <  0.50) and therefore, all variables remained in the modeling process. We then modeled all possible combinations of the five predictor variables. For the categorical variables of land cover and aspect, we used forested habitats and southerly aspect as a reference categories, both of which are commonly used by prey species.

We calculated Akaike’s Information Criterion adjusted for small sample size (*AICc*), ΔAICc, and Akaike weights (*w*_*i*_) for each model (Burnham & Anderson, 2002). We considered our top-ranked model and those models with ΔAICc values ≤2.0 to have provided the strongest empirical support for explaining variation in the locations where red foxes scavenged mountain lion kills; in cases where top models included nesting, we retained the simplest models to avoid the inclusion of uninformative parameters (Burnham & Anderson, 2002; Arnold, 2010). Lastly, we calculated evidence ratios for top models (Burnham, Anderson & Huyvaert, 2011) and assessed effect sizes of informative parameters by calculating Cohen’s *d* test statistics (difference between the means/pooled standard deviation). Statistics were conducted in R (R Core Team, 2017) with the lme4 (Bates et al., 2015) and MuMIn (Barton, 2016) packages.

## Results

### Mountain lion kill sites monitored and red fox scavenging

On average a camera remained at a kill site for 6.4 (±SD = 3.7) days in the winter and 6.1 (±3.3) days in the summer. Because we omitted mountain lion kills <25 kg from this analysis, we detected red foxes at 149 kills rather than the 151 documented in Elbroch et al. (2017). Foxes scavenged a greater proportion of winter kills than summer kills (70.3% in winter vs. 48.9% in summer; *z* = 3.291, *P* = 0.001), and on average scavenged longer at winter kills than summer ($\bar {X}=102.7\pm 138.3$ min for winter vs. $\bar {X}=39.7\pm 74.0$ for summer; *t*_135_ =  − 3.014, *P* = 0.003). When foxes were present, there were approximately 1.5 times more foxes per kill in winter than summer (1.83 vs. 1.16 foxes per kill, respectively; *t*_135_ = 5.94, *p* < 0.001).

Mountain lion presence influenced red fox scavenging in summer, but not in winter. Red foxes preferentially scavenged after the departure of the mountain lion in summer (20 kills while the mountain lion was still feeding from the carcass vs. 36 kills after the mountain lion had departed; ${\bar {X}}_{135}=11.25$, *P* = 0.001), but scavenged regardless of the presence of the mountain lion in winter (61 kills while the mountain lion was still feeding from the carcass vs. 65 kills after the mountain lion had departed; *χ*^2^_135_ = 0.25, *P* = 0.62). At kills at which foxes scavenged while mountain lions were present, foxes did not exhibit any changes in vigilance after the mountain lion departed (*t*_63_ = 5.94, *P* = 0.80).

### Spatial patterns of red fox scavenging

Winter mountain lion kills were more aggregated than summer kills (*t*_98_ = 4.17, *P* < 0.001). In winter, kills were 456.6 (CIs [361.5–551.8]) m apart, and in summer, 1007.8 (CIs [762.4–1253.3]). Our analyses also identified different spatial attributes of red fox scavenging, dependent upon season, though these patterns were weak overall. Two top models provided insights into winter patterns (Table 1). The top-ranked model received 2.7 times more empirical support than our second model based on an evidence ratio, and emphasized variation in distance to forest edge (*β* = 0.473, SE = 0.263) in locations where red foxes were scavenging mountain lion kills versus where they were not. Nevertheless, this effect was small (Cohen’s *d* = 0.34). Our second-ranked model contributed insights absent in our top model, and showed that red foxes were more likely to be detected at higher elevations than lower (*β* = 0.279, SE = 0.194). Again, this effect was small (Cohen’s *d* = 0.27). Our null model, which only included the intercept and random effect (Table 1), was only 2.01 ΔAICc from the top-ranked model, emphasizing the fact that the covariates we tested in our models little influenced the spatial patterns of red fox scavenging in winter.

**Table 1:** The top 10 models predicting where red foxes scavenged at mountain lion kill sites, estimated with generalized linear mixed models, during winter and summer in the southern Greater Yellowstone Ecosystem from 2012–2015.

Model		*df*	Log-likelihood	*AICc*	Δ*AICc*,	*w*_*i*_	Removed due to nesting
Winter							
1	Edge	3	−81.905	170.000	0.000	0.257	
2	Edge + Elevation	4	−81.114	170.500	0.540	0.196	^a^
3	Elevation	3	−82.895	172.000	1.980	0.096	
4	Null	2	−83.958	172.000	2.010	0.094	
5	Ruggedness + Edge	4	−81.902	172.100	2.120	0.089	^a^
6	Ruggedness + Edge + Elevation	5	−81.100	172.700	2.660	0.068	^a^
7	Ruggedness	3	−83.821	173.800	3.830	0.038	
8	Ruggedness + Elevation	4	−82.870	174.000	4.050	0.034	^a^
9	Habitat + Edge + Elevation	7	−79.883	174.600	4.640	0.025	^a^
10	Habitat + Edge	6	−81.159	175.000	4.970	0.021	
Summer	Null						
1	Elevation	3	−62.973	132.200	0.000	0.268	
2	Edge + Elevation	4	−62.914	134.300	2.070	0.095	
3	Ruggedness + Elevation	4	−62.938	134.300	2.110	0.093	
4	Null	2	−65.112	134.400	2.140	0.092	
5	Habitat	5	−62.247	135.200	2.960	0.061	
6	Edge	3	−64.504	135.300	3.060	0.058	
7	Habitat + Elevation	6	−61.186	135.300	3.130	0.056	
8	Ruggedness	3	−65.080	136.400	4.220	0.033	
9	Ruggedness + Edge + Elevation	5	−62.891	136.500	4.250	0.032	^a^
10	Elevation + Aspect + Elevation	6	−61.757	136.500	4.270	0.032	

**Notes.**

a Indicates the model was removed due to nesting.

For summer, our analysis identified one top model that accounted for 26.8% of model weights (Table 1), and emphasized variation in the elevation at which foxes were scavenging. In contrast to winter, foxes were more likely to occur at mountain lion kills at lower elevations relative to where mountain lions kills occurred across the landscape (*β* =  − 0.472, SE = 0.245). This effect was small, approaching medium (*d* = 0.41). As during winter, the null model performed well, only 2.14 ΔAICc from the top-ranked model (Table 1), indicating that red foxes were not strongly selecting for any spatial attributes of mountain lion kills at which to scavenge.

## Discussion

In support of our hypotheses, red foxes benefitted from carrion provided by mountain lions throughout the year, but utilized carrion resources more in winter than summer. We found mixed support for our hypothesis that foxes would wait for mountain lions to depart carcasses before scavenging, dependent upon season, and our results failed to support our hypothesis that foxes would exhibit higher vigilance when scavenging a carcass while the mountain lion was nearby. Red foxes were the most common scavenger detected at mountain lion kills in our study area (Elbroch et al., 2017) (Fig. 2), feeding at more kills than common scavengers like black-billed magpies (*n* = 149) and ravens (*n* = 59). Our results failed to support our hypothesis that red foxes would preferentially scavenge at kills near forest edges or structured habitat; instead they scavenged from kills across all habitat types. As predicted, fox scavenging was not affected by aspect, terrain ruggedness or elevation. Foxes exhibited little selection for specific spatial attributes of mountain lion kills at which to feed, as indicated by low effect sizes (Cohen’s *d*) for significant covariates in top models and the comparable performance of our null models to top models.

Red fox scavenging behaviors varied with season. In winter, foxes utilized a greater percent of mountain lion kills, scavenged in greater numbers, and scavenged for longer durations. These differences highlight the importance of carrion resources to red foxes in winter, a pattern highlighted by several other studies as well (Cagnacci, Lovari & Meriggi, 2003; Needham et al., 2014; Carricondo-Sanchez et al., 2016). The question, then becomes, why do red foxes scavenge more from mountain lion kills in winter than in summer?

We suspect that red foxes scavenged more often from mountain lion kills in winter to cope with resource shortages. Cold winter temperatures and heavy snowfall lead to resource scarcity in temperate systems, and many animals cope with seasonal scarcities by hibernating through the scant period or alternatively, adaptive foraging strategies like food hoarding (Vander Wall, 1990). Carrion produced by large predators can buffer resource scarcity for medium-sized carnivores that do not hibernate, including red foxes, and increase fitness for medium and small carnivores in areas with severe winters (Selva et al., 2005; Pereira, Owen-Smith & Moleón, 2014). Red fox scavenging may have also increased in winter because mountain lion kills were more aggregated in this season, allowing subordinate species to exploit these resources more successfully (Wilmers et al., 2003a). Competition for carrion may have also decreased for foxes in winter, because competitively-dominant bears were hibernating in this season. Gray foxes (*Urocyon cinereoargenteus*) were limited by black bears at carcasses in a Mediterranean system (Allen et al., 2015).

Competition at and near carrion structures mammalian communities (Cortés-Avizanda et al., 2009; Allen et al., 2015), but if abundant, carrion may also buffer the impacts of competition, allowing subordinate species like red foxes to coexist with dominant species (Sivy et al., 2017). Red foxes are subordinate to numerous carnivores, including coyotes, wolves, bears, and mountain lions. In North America, research has shown that red fox abundance and behavior are generally limited by coyotes, which in turn are limited by wolves (Levi & Wilmers, 2012; Newsome & Ripple, 2015) and potentially mountain lions (Elbroch & Kusler, 2018). Future research needs to determine whether mountain lions act as shields that protect foxes from coyotes, and provide foxes access to resources that otherwise might be secured by coyotes and other dominant scavengers; mountain lion presence may allow foxes to coexist at higher densities with coyotes than in their absence. We also need to determine whether seasonal variation in the spatial distributions of carrion benefits fox versus coyote exploitation, and whether the pattern of carrion availability influences coexistence with coyotes at higher numbers of foxes.

**Figure 3:**
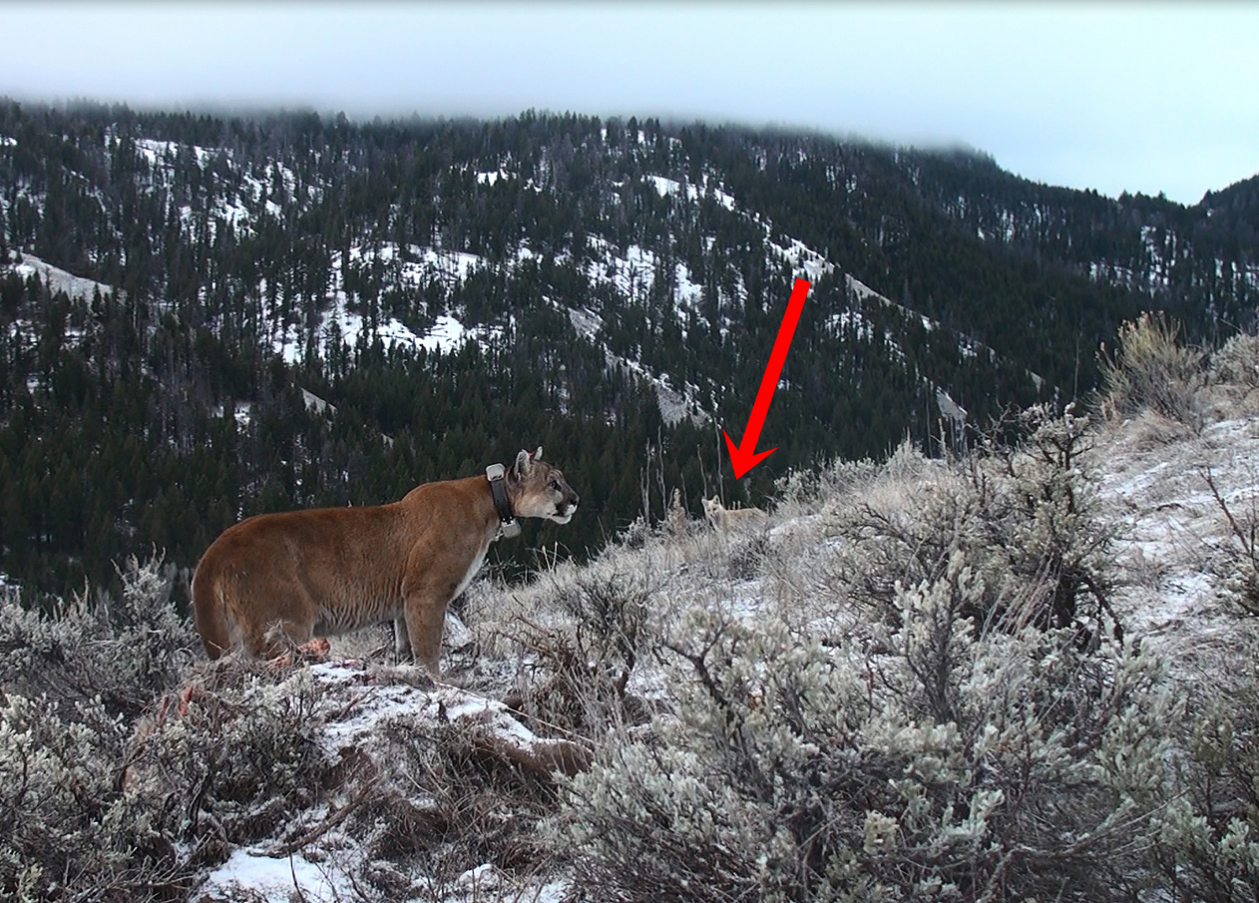
A red fox awaiting the departure of a mountain lion so it can feed at the kill. Despite occasionally falling prey to mountain lions, red foxes often fed in close proximity to the large predator, especially during winter months. Photograph by L Mark Elbroch.

Red foxes also varied whether they scavenged while the mountain lion was present depending upon season. In summer, foxes more often scavenged kills after the mountain lion had departed the site, whereas in winter, red foxes were detected while the mountain lion was present as often as after the large predator had departed. We propose two biological explanations for this seasonal variation in behavior. First, red foxes may detect mountain lion kills faster in winter than in summer. We speculated that red foxes may even follow mountain lions in winter, as arctic foxes follow polar bears and wolves (Chesemore, 1968), though we lacked the movement data of foxes to test this hypothesis. Second, red foxes may be hungrier in winter and therefore take greater risks, more frequently scavenging in the presence of a nearby predator (Fig. 3). Hunger increases risk-taking in foraging vertebrates (Mathot et al., 2015; Blecha, Boone & Alldredge, 2018).

## Conclusions

Our research highlighted the ecological relationship between red foxes and mountain lions in the GYE. Our results showed that red foxes scavenged mountain lion kills more often in winter, fed for longer in winter, and that on average, a greater number of foxes fed at each carcass in winter. Further, foxes fed more often when the mountain lion was present in winter. Counter to our hypotheses, foxes did not preferentially scavenge after the mountain lion had departed the site, nor did they exhibit greater vigilance behavior when feeding while the mountain lion was nearby. This suggest that food availability may be a more powerful influence on fox foraging decisions than the potential risks of intraguild predation.

Mountain lions tolerate high levels of scavenging (Elbroch et al., 2017), thus the intensity of red fox scavenging at their kills may not impact mountain lion fitness, and instead facilitate the dispersion of beneficial carrion resources created by this apex predator. Large carnivores, and mid-trophic felids like mountain lions in particular, are essential producers of carrion vital to biodiversity and ecosystem health (Wilson & Wolkovich, 2011; Moleón & Sánchez-Zapata, 2015; Inger et al., 2016; Elbroch et al., 2017). In turn, scavengers play critical roles in distributing these resources and increasing the heterogeneity of resources that support biodiversity and ecosystem structure (Bump, Peterson & Vucetich, 2009; Cortés-Avizanda et al., 2009; Barton et al., 2013; Allen et al., 2015), as well as ecological resilience (DeVault, Rhodes Jr & Shivik, 2003; Wilson & Wolkovich, 2011; Moleón et al., 2014).

##  Supplemental Information

10.7717/peerj.5324/supp-1 Supplemental Information 1 Red foxes scavenging at mountain lion kills. Video by Mark Elbroch

10.7717/peerj.5324/supp-2 Supplemental Information 2 Raw data of mountain lion kill site video database Database used to catalog video footage from mountain lion kill sites.

## Additional Information and Declarations

### Data Availability

The following information was supplied regarding data availability: The raw data are provided in a Supplemental File.
